# A DNA-binding-site landscape and regulatory network analysis for NAC transcription factors in *Arabidopsis thaliana*

**DOI:** 10.1093/nar/gku502

**Published:** 2014-06-09

**Authors:** Søren Lindemose, Michael K. Jensen, Jan Van de Velde, Charlotte O'Shea, Ken S. Heyndrickx, Christopher T. Workman, Klaas Vandepoele, Karen Skriver, Federico De Masi

**Affiliations:** 1 Department of Biology, University of Copenhagen, 2200 Copenhagen, Denmark; 2 Novo Nordisk Foundation Center for Biosustainability, Technical University of Denmark, DK-2970 Hørsholm, Denmark; 3 Department of Plant Systems Biology, VIB, 9052 Ghent, Belgium; 4 Department of Plant Biotechnology and Bioinformatics, Ghent University, 9052 Ghent, Belgium; 5 Center for Biological Sequence Analysis, Institute for Systems Biology, Technical University of Denmark, 2800 Kgs. Lyngby, Denmark

## Abstract

Target gene identification for transcription factors is a prerequisite for the systems wide understanding of organismal behaviour. NAM-ATAF1/2-CUC2 (NAC) transcription factors are amongst the largest transcription factor families in plants, yet limited data exist from unbiased approaches to resolve the DNA-binding preferences of individual members. Here, we present a TF-target gene identification workflow based on the integration of novel protein binding microarray data with gene expression and multi-species promoter sequence conservation to identify the DNA-binding specificities and the gene regulatory networks of 12 NAC transcription factors. Our data offer specific single-base resolution fingerprints for most TFs studied and indicate that NAC DNA-binding specificities might be predicted from their DNA-binding domain's sequence. The developed methodology, including the application of complementary functional genomics filters, makes it possible to translate, for each TF, protein binding microarray data into a set of high-quality target genes. With this approach, we confirm NAC target genes reported from independent *in vivo* analyses. We emphasize that candidate target gene sets together with the workflow associated with functional modules offer a strong resource to unravel the regulatory potential of NAC genes and that this workflow could be used to study other families of transcription factors.

## INTRODUCTION

Plants use cellular strategies to survive exposure to biotic and abiotic stresses. Drought, salt, high temperature and microbial infections are amongst the most frequent abiotic and biotic stresses encountered by plants (1–4) Expression of genes that function in stress sensing and tolerance are regulated upon stress exposure by specific transcription factors (TFs) (1,2). The NAC (NAM/ATAF/CUC) family of proteins is a major group of plant-specific TFs involved in plant development, senescence, secondary cell wall formation and stress responses (5–7). The well-studied model plant *Arabidopsis thaliana* and economically important crops such as *Nicotiana tabacum*, *Hordeum vulgare* and *Oryza sativa* each hold the potential to express more than 100 different NAC proteins (2,5,6). When genes encoding NAC TFs are over-expressed in plants, robust phenotypes including salt and drought tolerance have been observed (2,8,9). Likewise, *nac* mutant plants have been shown to display loss of secondary wall thickening, perturbed resistance towards microbial attack as well as delayed senescence (1,5,6,10), though functional redundancy often has hampered characterization of individual NAC members.

NAC proteins consist of a conserved N-terminal deoxyribonucleic acid (DNA) binding domain (DBD), known as the NAC domain, which is also responsible for the oligomerization into dimeric proteins (7,11). The C-terminal region of NAC members is more diverse, intrinsically disordered, and functions as a transcription regulatory domain (12,13). Determination of the X-ray structure of the NAC domain from *A. thaliana* ANAC019 revealed a novel dimeric DBD predominantly composed of β-sheets with no well-characterized DNA-binding motifs (11). Characterization of the dimerization surface demonstrated that ANAC019 is only able to bind DNA as homo- and hetero-dimers (7). In addition, the consensus DNA-binding sequences of two distantly related NAC TFs, ANAC019 and ANAC092, were identified by *in vitro* selection (SELEX) and appeared to have minor differences in their DNA-binding specificities (7). For both proteins, the identified core consensus DNA-binding sequence was TTNCGT[G/A]. Interestingly, in a recent study it was found that nine distantly related NAC TFs were able to bind this sequence, though with different affinities (13). In line with these results, it has been shown that several other NAC TFs bind the core CGT[G/A], but with considerable sequence differences in the flanking bases of the binding site (14). Thus, the flanking bases next to the core CGT[G/A] of NAC binding sites (NACBSs) in promoters may determine the binding specificities and fine-tune affinity for different NAC TFs *in vivo*. This effect was recently demonstrated to be highly relevant in the family of basic helix-loop-helix (bHLH) transcription factors (15–17).

Apart from focused dimerization and DNA-binding studies on NAC TFs, global mapping of gene regulatory networks (GRNs) can be facilitated by high-throughput approaches that allow for the discovery and high-resolution characterization of genome-wide DNA-binding specificities of DNA-binding proteins. Protein binding microarrays (PBMs) have been widely used as an unbiased and condition-independent method for the identification of high-resolution DNA specificities for a larger number of TFs from several organisms (15,18–20). PBMs can uncover binding specificities of TFs at the k-mer level, with single-base resolution. Also, PBM data have been shown to strongly correlate with surface plasmon resonance studies of TF–DNA interactions (21,22), thus allowing the use of PBM data to analyse biologically relevant data. Further integration of such data with genome annotations, gene expression data and functional modules (23) will result in the functional characterization of the mapped observed TF–DNA interactions and possibly the unravelling of TF and condition-specific GRNs (12,13,15,24,25).

In this study, we report the integration of PBM results with co-expression data and functional module enrichment to outline the regulatory network for 12 NAC proteins. Furthermore, we show that this integrative strategy, applicable to any TF target gene analysis, allows for the refinement and increase in significance of TF target genes. We also use our PBM data to motivate mutations in an element identical to a region of a selected target gene promoter and propose that a simple 2-nucleotide substitution may be exploited to control binding of native TFs to novel promoter elements. Finally, co-expression analysis is used to validate the regulatory potential predicted from our unbiased PBM analysis. This study is the first systems-wide analysis of the NAC family of transcription factors resulting in a global map of the NAC DNA-binding specificities in *A. thaliana* and we envision the data to be useful for future engineering of improved stress responses in plants.

## MATERIALS AND METHODS

### Sequence analysis of the NAC family

Multiple alignments, phylogenetic tree and the sequence similarity matrix of the DBDs of all proteins were generated using ClustalW (26) and drawn using MatLab (Mathworks, Natick, MA, USA). BoxShade (http://www.ch.embnet.org/software/BOX_form.html) was then used for producing graphical representations of the multiple alignment.

### Cloning and recombinant protein production

Oligonucleotides, restriction enzymes and vectors used for cloning of Glutathione-S-Transferase (GST) tagged proteins analysed in this study are listed in Supplementary Table S1. Cloning and production of several of the GST-recombinant proteins have already been described (13). In addition, cDNA clones acquired from the Arabidopsis Biological Resource Center were amplified by polymerase chain reaction (PCR) to obtain the region encoding the NAC domain of ANAC055, ANAC072, NAP and NST2, full-length ANAC092 and the DBD of WRKY1 (27). Finally, the NAC domain encoding region of SND1 was synthesized (Eurofins MWG Operon) and used for PCR. The PCR products were inserted into the vectors as shown (Supplementary Table S1). For the zinc-finger TFs VOZ2 and WRKY1 50-μM zinc acetate was added to the growth medium. After induction, cells were harvested and sonicated and GST-tagged proteins were purified on glutathione–Sepharose 4B resin (GE Healthcare) as described (13). Purified recombinant proteins were analysed by sodium dodecyl sulphate-polyacrylamide gel electrophoresis and absorbance scans. Protein concentrations were estimated from A_280_ measurements. By using this procedure highly pure GST-tagged recombinant proteins were produced and no further purification was needed. A subset of the NAC proteins described above was also produced by PURExpress *In Vitro* Protein Synthesis transcription/translation kits (New England Biolabs) according to the manufacturer's instructions. The concentration of purified GST-tagged proteins was quantified by western blotting using anti-GST antibody (Invitrogen) by comparison to a dilution series of recombinant glutathione-S-transferase (Sigma).

### PBM experiments and data analysis

Oligonucleotide arrays were made double-stranded by primer extension and PBM experiments were performed as described previously using custom ‘all 10-mer’ array design using the Agilent ‘4 × 180K’ array format (Agilent Technologies, Inc.) (28). All PBM experiments were performed in duplicate at a final protein concentration between 200 and 500 nM. Microarray scanning, spot quantification, data filtering, normalization and primary analysis were performed as previously described (15,28). Significant k-mers were selected by identification of all words showing an Enrichment Score (ES) equal to or greater than 0.4 for at least one studied TF. Contrary to other similar studies, we here retrieved all gaped or un-gaped 8-mers resulting in a final set of 4821 significant k-mers (Supplementary Table S2).

**Figure 1.**
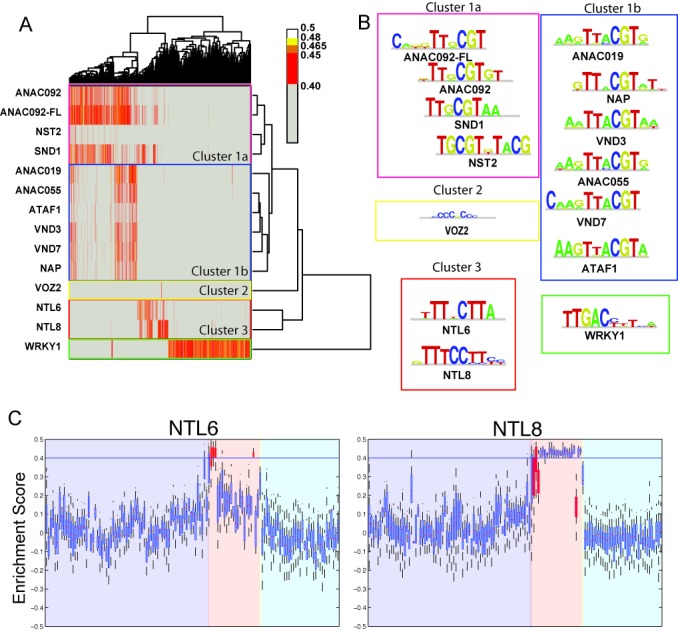
DNA-binding profiles of NAC TFs can be separated into five specificity clusters. (**A**) Bi-dimensional clustergram of the identified 4821 significant k-mers (X-axis) versus studied TFs (Y-axis). Internal rectangles indicate clusters of TFs showing similar DNA specificity profiles at the k-mer level. (**B**) DNA specificities for each TF, grouped in clusters as in (A). (**C**) Enrichment Score distributions for NTL6 and NTL8 shown as boxplots. Dark filled boxes show NTL6 specific k-mer groups. The identity of each k-mer is available in Supplementary Figure S3B. For each box, the central mark represents the median value for the distribution, the box edges represent the 25th and 75th percentiles and the whiskers extend to the last non-outlier data point, as described in Matlab's ‘boxplot’ help documentation (http://www.mathworks.se/help/stats/boxplot.html).

‘Core words’ used for boxplots were identified by a combination of a statistical method (‘preferred k-mers’) from Jiang *et al.* (29) and a visual approach of the previously described matrix. This resulted in the identification of 130 core words of length 6 or 7 that are able to describe the exact specificities of each TF. PWM logos were drawn using the enoLogos engine (30). Heatmap figures were made using Matlab (Figure 1) and Genesis (31) (Supplementary Figures S6 and S8).

### Detection of target genes, integration of co-expression information, gene function enrichment analysis and motif conservation

Target genes were predicted by initially determining for each TF a set of high scoring seed 8-mers (ES > 0.45) and mapping these to the promoters of all *Arabidopsis* genes (TAIR10). A promoter was defined as the 1000 bp upstream of a gene or a shorter region if the adjacent upstream gene is located within a distance smaller than 1 kb.

To refine the set of PBM-predicted (P) target genes, expression data were integrated to define target genes that are also co-expressing with other predicted target genes (P+COE). Based on 14 Affymetrix ATH1 microarray expression compendia delineated by De Bodt *et al.* (32), we defined for each gene a co-expression cluster by selecting the top-100 co-expressed genes based on Pearson correlation coefficients. A target gene was retained as P+COE target if its co-expression cluster was enriched for target genes of the same TF (hypergeometric distribution, *P*-value < 0.05).

To evaluate the evolutionary conservation of individual k-mer instances, a multi-species phylogenetic footprinting approach was applied. For each Arabidopsis target gene the orthologous genes from 11 other dicot species [*Malus domestica, Fragaria vesca, Manihot esculenta, Medicago truncatula, Carica papaya, Glycine max, Lotus japonica, Ricinus communis, Theobroma cacao, Populus trichocarpa and Vitis vinifera*; source PLAZA 2.5 (33)] were retrieved using the PLAZA Integrative Orthology method. First, the 1-kb orthologous promoter sequences were aligned to the query promoter using the Sigma alignment tool (34). Next, all pairwise alignments for each query gene were aggregated on the query sequence generating a multi-species conservation plot that shows for each nucleotide of the investigated region how many species support this nucleotide through pairwise footprints. All footprints for each level of conservation were extracted from the multi-species conservation plot. Finally, the significance of the observed multi-species footprints, per Arabidopsis target gene, was determined by randomly sampling 1000 non-orthologous gene sets, maintaining the gene and species composition as observed in the real orthologous data set and scoring in how many random gene sets a footprint with a similar or better multi-species conservation was found. Footprints with a false discovery rate <5% were used to identify conserved PBM motif instances. The significance of the overlap was calculated using the hypergeometric distribution (*P*-value <0.05). Fold enrichment was calculated using the formula (*k*/*n*)/(*K*/*N*), where *k* is the number of recovered differentially expressed (DE) genes within the predicted target genes, *n* is the number of predicted target genes, *K* is the number of DE genes and *N* is the number of genes in the genome.

### Construction and biological evaluation of the NAC GRN

In order to construct a GRN all P+COE target genes of all TFs were used. In order to evaluate the function of these P+COE target genes, we determined, per TF, enriched functional modules for all target genes. The associated Gene Ontology (GO) terms of each enriched functional module were mapped to their parental GO terms, GO slim terms were selected and these GO slim terms were grouped into 10 functional categories. In order to obtain functional categories, all GO slim terms were clustered on their enrichment in functional modules and groups of GO slim terms that clustered together were isolated as categories (tropism: tropism; cellular homeostasis: cellular homeostasis; stress cell death and signalling: cell–cell signalling, regulation of gene expression, epigenetic, response to stress, response to biotic stimulus, response to abiotic stimulus, death, cell death, response to external stimulus, cell communication, response to extracellular stimulus; transport: transport; signal transduction and response to endogenous stimulus: signal transduction, response to endogenous stimulus; catabolic process: catabolic process; energy lipid carbohydrate and secondary metabolism: generation of precursor metabolites and energy, photosynthesis, lipid metabolic process, carbohydrate metabolic process, secondary metabolic process; cell cycle: cell cycle; translation and protein metabolism: translation, protein metabolic process; growth reproduction and development: reproduction, multicellular organismal development, anatomical structure morphogenesis, embryo development, post-embryonic development, fruit ripening, abscission, pollination, pollen–pistil interaction, flower development, cellular component organization, cell growth, cell differentiation, growth). The network depicted in Figure 3 was constructed using the Node Chart Plugin for Cytoscape 2.8.2 (35). Only modules with enriched GO slim terms are depicted. This plugin allows for a module node to be used as a pie chart and through colour-coding for the different functional categories, this allowed visualizing the predicted functional role of each module associated with each TF.

**Figure 2.**
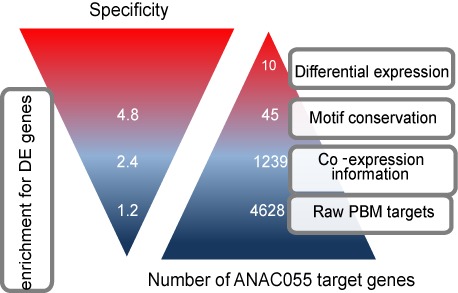
Overview of additional genomic filters leading to TF target genes with increased biological relevance. Starting from the predicted PBM ANAC055 target genes, the inclusion of co-expression information and motif conservation leads to a reduced set of target genes (right triangle) showing increased enrichment for DE genes obtained from an ANAC055 perturbation transcript profiling experiment (left triangle). Specificity refers to the enrichment fold for DE genes in the different target gene sets. Whereas motif conservation results in an increased specificity for DE genes compared to predicted PBM targets for ANAC019, ANAC055 and ANAC092, combining co-expression information with motif conservation leads to an additional gain in enrichment for ANAC055.

**Figure 3.**
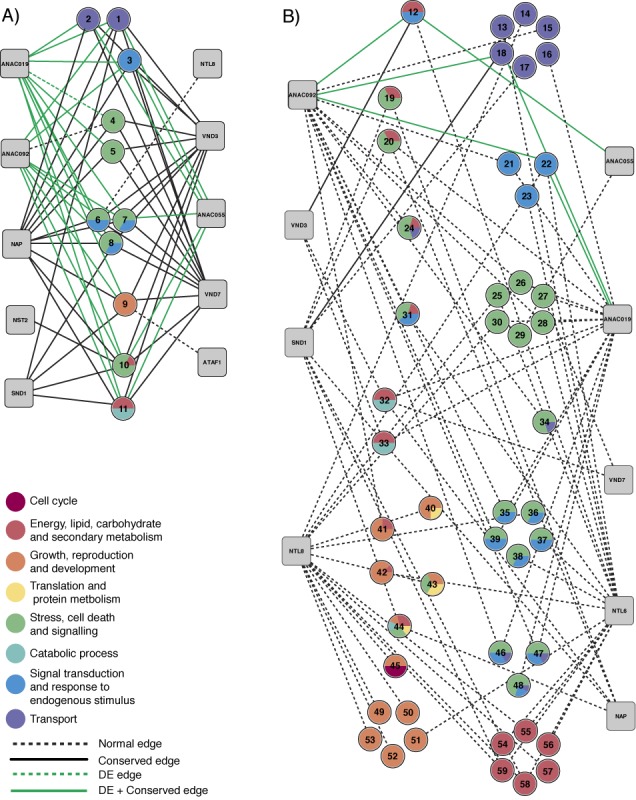
Functional overview of modules enriched for TF-target genes. Panel (**A**) shows the TF module network for enriched modules that are shared between five or more TFs whilst panel (**B**) shows the TF module network for enriched modules that are shared between less than five TFs. Grey boxes represent TFs whilst coloured circles refer to modules attributed to different functional categories. The numbers in the coloured circles refer to the functional gene modules described in Supplementary Table S6. Whereas solid grey edges denote module enrichment for candidate PBM target genes, solid black lines indicate that a DE gene for that TF is present in the module. Arrowed lines denote candidate target genes with a conserved motif.

### Electrophoretic mobility-shift assay

Purified GST-ANAC092(1-176) and GST-NTL6(1-168) were tested for functionality in electrophoretic mobility-shift assays (EMSAs) using a ^32^P-labelled double-stranded oligonucleotide of the palindromic NACBS [PalNACBS; Supplementary Table S1; (7)], the wild-type MYB90 promoter fragment (Supplementary Table S1; WT promoter) and the synthetic promoter fragment (Supplementary Table S1; Synthetic promoter). EMSAs were performed as described previously (7,36). The oligonucleotides used in EMSA were initially pairwise annealed in 100 μl (20-mM Tris-HCl, pH 8.0, 20-mM MgCl_2_) by heating the solution to 95**°**C for 5 min followed by slowly cooling to room temperature, which normally takes hours. Small aliquots were then taken out when needed for labeling, purification and finally EMSA. The DNA concentration in EMSA was kept at 75 pM, which is roughly 1000-fold lower than the estimated Kd for the interaction (36).

### Co-expression analyses

To investigate if genes differentially expressed in *anac092* mutant compared to wild-type Col-0 plants maintain expression perturbations during environmental conditions known to affect *ANAC092* levels, we data-mined >3.000 Col-0 wild-type ATH1 microarray samples from the Genevestigator data repository (37). Using a stringent (>2-fold regulation; *P* < 0.05) selection criterion for *ANAC092* transcript level perturbations, we found 705 microarray slides from 160 perturbations. This data set was used to perform hierarchal clustering (euclidian distance) of *ANAC092* and 107 putative target genes differentially expressed in *anac092* mutant compared to wild-type Col-0 plants, all containing ANAC092 BS in their 1-kb promoter.

## RESULTS

### DNA-binding specificity analysis of individual NAC TFs

Systematic analysis of NAC DNA-binding specificity by PBMs (15,18,28) was performed on 12 NAC TFs representing functionally important clades and spanning the phylogenetic diversity of the NAC family (Supplementary Figure S1 and Supplementary Document S1) (13). ANAC019 was selected because its NAC domain structure is known (11,36) and because it is implicated in networks of stress responses and senescence (13,38). ANAC055 and ATAF1 are closely related to ANAC019 (13,39), and ATAF1 is a control for the PBM experiments (40). They all cluster together with senescence-associated NAP (41) based on hormone-dependent gene regulation (42). Therefore, analysis of these NAC TFs could reveal simple relationships between amino acid sequence and DNA-binding specificity. ANAC092/ORE1 represents a functionally important NAC sub-group (13,43). VND3, VND7, NST2 and SND1 represent an NAC sub-group that is central to secondary cell wall formation (44–46). NTL8 and NTL6 are transmembrane NAC TFs (47), and NTL6 acts through known binding sites in ‘pathogenesis-related (PR)’ genes (48) allowing comparison of PBM and *in vivo* promoter binding data. The distant NAC members, SOG1 (49), ANAC003 (13) and VOZ2, were also included. VOZ2 has a zinc-finger region N-terminally of the NAC domain (50). In the other NAC TFs, the N-terminal NAC domain is followed by various intrinsically disordered transcriptional regulatory domains (13) (Supplementary Figure S2). Since only the NAC domain is used in this study and since remote disordered regions may fine-tune both specificity and affinity of DNA binding (51), full-length ANAC092 was also used for the PBM experiments. Finally, the WRKY domain of the WRKY1 TF was included due to its well-defined DNA-binding specificity (52).

We generated a list of 4821 gaped and ungaped 8-mers (the Materials and Methods section and Supplementary Table S2) that showed an ES equal to or greater than 0.40 for at least one tested protein. Clustering of these k-mers revealed that NAC transcription factors can be separated into three distinct groups characterized by their DNA specificities (Figure 1A). Interestingly, these groups largely match the three main branches in the phylogenetic tree shown in Supplementary Figure S1A.

Cluster 1, which comprises ANAC019, ANAC055, ANAC092, ATAF1, NAP, NST2, SND1, VND3 and VND7, shows a clear binding preference for the accepted NAC-BS model, T[G/A]CGT (Figure 1B) (53). This cluster can be further separated into clusters 1a and 1b. Cluster 1a contains ANAC092, SND1 and NST2, which show a distinctive specificity for TTGCGT. Cluster 1b contains ANAC019, ANAC055, VND7, ATAF1, NAP and VND3, which show a main specificity for the TACGT core motif (Figure 1B). This agrees with our earlier results on ATAF1 using a different set of deBruijn sequences and array design (40). Interestingly, VND3 and VND7 are closer, in their sequences, to proteins in cluster 1a (Supplementary Figure S1A) yet their DNA specificity model groups these TFs with cluster 1b hinting at minor, yet critical, residue differences that would be able to dictate DNA-binding properties of the TF.

Reassuringly, cluster 1a also contained both forms of ANAC092. This observation, together with the logos for both proteins in Figure 1B, shows that the full-length version of ANAC092 binds with higher affinity to an expanded range of k-mers compared to the NAC DBD-only version. Importantly, the DNA-binding specificity was not significantly changed by the disordered C-terminus of ANAC092 (Figure 1B and Supplementary Figure S2). This suggests that the intrinsically disordered region of ANAC092 assists the DNA binding giving an overall better binding/higher affinity, possibly through modulation of conformation, flexibility or spacing within the DNA–protein complex (51).

Cluster 2 only contains VOZ2, whose distinct preference has a very strong resemblance to a zinc-finger motif CCCGCC as shown by, for example, Klf7 (19) or Sp1 (54). It has been shown that VOZ2's zinc finger is required for DNA binding and this specificity could confirm this requirement (50). SOG-1 and ANAC003 failed to generate binding data.

Cluster 3, containing NTL6 and NTL8, shows a surprising specificity for k-mers containing TT(A/C/G)CTT (Figure 1B) and, additionally, NTL6 and NTL8 specific k-mers do not appear to show any overlap (Figure 1A) with cluster 1a, 1b or 2. Finally, our PBM data confirm the specificity of WRKY1 for the W box consensus motif TTGACC/T (Figure 1A), as previously reported from *in vivo* Chromatin Immunoprecipitation (ChIP) studies (52).

From our PBM analysis, we conclude that NAC proteins show specificities for at least three different consensus models and that the differences in DNA-binding specificities largely match the three main branches in the phylogenetic tree shown (Supplementary Figure S1A). This indicates that NAC DNA-binding specificities may be estimated from their DBD's sequence.

In order to uncover hidden specificities present in each TF's data, we analysed the available PBM data using shorter word sequences that can represent the full extent of the data in a simple manner. Using a combination of manual and statistical analyses (29) we identified 130 6-mers (i.e. ungaped 6-mers and gaped 7-mers) that are able to describe, with high precision, the variation in specificities for each TF at a single-base resolution. Additionally, these k-mers allow for the direct comparison of the differences in relative affinity of each protein for each k-mer. Analyses of these comparisons (Figure 1C and Supplementary Figure S3) result in the identification of TF-specific k-mers and in a high-resolution fingerprint of the relative affinities of each protein against each key k-mer. For example, NTL6 and NTL8 show similar overall specificity models (Figure 1B), yet it is evident that their binding preferences, when looking at shorter k-mers, are dramatically different (Figure 1C) and there is no overlap between high-ES k-mers for NTL6 and NTL8, even though their overall specificity models are very similar (Figure 1B). Finally, we can rank the individual TFs by overall DNA-binding specificity. By simple observation of the boxplots in Supplementary Figure S3, we can conclude that ANAC019, ANAC055, ANAC092, SND1 and NTL8 show broad and high specificities, within their subclass (or cluster) compared to the other NACs.

Our results show that though some NAC TFs share specificities, evident differences amongst top-ranking k-mers are observed in their binding site preferences. Thus from this detailed analysis we can generate precise specificity models, or fingerprints, for each TF which will uniquely define the spectrum of DNA sequences recognized by each NAC protein.

### Identification of direct NAC target genes from DNA-binding data and microarray analysis

Using our PBM results, we next aimed at determining target genes involved in NAC-specific signalling in Arabidopsis. Raw PBM target genes were predicted by initially determining, for each TF, a set of high scoring seed 8-mers and mapping these to the 1-kb promoters of all *Arabidopsis* genes. This resulted in a large number of predicted target genes (P) for the different TF (Supplementary Table S3 and Supplementary Figure S4).

For three TFs (ANAC019, ANAC055 and ANAC092), transcriptional profiling of mutant lines resulted in a set of DE genes (38,43), which were used to evaluate our data processing methodology and to define additional criteria to delineate functional target genes. Although DE genes contain directly as well as indirectly regulated genes, they offer a valuable source of information to assess whether TF binding inferred through PBMs corresponds with TF regulation. As the sets of P target genes showed only moderate enrichment for DE genes in the mutant lines (1.09–1.21-fold enrichment) (Supplementary Figure S5), co-expression and motif conservation information were combined with the PBM data to identify more biologically relevant target genes. Integration of expression data, through enrichment analysis of gene-centric co-expression clusters for P target genes (see the Materials and Methods section), resulted in a reduced set of predicted + co-expressed PBM target genes (P+COE) (Figure 2). For all three PBM experiments these candidate target gene sets showed significant overlap with the DE genes yielding higher enrichments (1.68–2.97-fold enrichment) compared with the full set of predicted target genes defined without co-expression information (Supplementary Figure S5). Conservation of PBM motif instances was determined using a multi-species alignment-based phylogenetic footprinting approach with 11 related dicotyledonous species (see the Materials and Methods section). The inclusion of motif conservation returns a set of target genes conserved within dicot plants (conserved P+COE), for ANAC055 these conserved targets showed an increased enrichment for DE genes (4.78-fold enrichment; see Figure 2) compared to only using co-expression as a filter. A similar increase in specificity for functional GO enrichments was observed when comparing the DE gene sets with subsequent filtering of the P target genes using co-expression and motif conservation (data not shown). These results demonstrate that the developed methodology combined with the application of complementary functional genomics filters makes it possible to translate, for each TF, the high-scoring k-mers into a set of high-quality predicted genes, which provide the basis to study different biological processes controlled by several NAC genes. All further analyses are performed using the P+COE target genes because this set has the best balance between sensitivity and specificity. The NAC P+COE target genes were used to generate a GRN comprising 22 489 interactions for 12 TFs and 9706 P+COE target genes (Supplementary Tables S3 and S4). A set of known TF–target gene interactions curated from literature (55) was used to evaluate the GRN. Experimentally determined target genes were present for three TFs (SND1, VND7 and NST2) in our study. Overall, 32% (31/98) of the interactions compiled from different small-scale experiments were recovered by our GRN, indicating that apart from generating many novel interactions, also multiple known interactions were successfully recovered using our approach. Condition- and tissue-dependent regulation, lack of co-factor data as well as chromatin state/accessibility information are factors that can interfere with the accurate detection of functional target genes and can cause the mis-identification of a limited set of known regulated genes.

To study the overlap of the P+COE target genes, the sets of target genes for the different TFs were compared (see Supplementary Figure S6 and Supplementary Table S5). Clustering of the TFs based on the shared target genes revealed two clusters, one containing ANAC092, NST2, ANAC019, ANAC055, NAP, ATAF1, VND3 and VND7, and one containing SND1, NTL6 and NTL8. Due to the low number of candidate target genes, VOZ2 shows very low overlap scores with the other TFs (Supplementary Figure S6). The high overlap scores between ANAC092, ANAC055 and ANAC019 (>5-fold enrichment, hypergeometric *P*-value <0.01) are in agreement with the significant overlaps between the DE genes obtained from transcript profiling on the corresponding mutants (3–6-fold enrichment, *P*-value <0.01; see Supplementary Figure S7), suggesting substantial functional redundancy between those TFs. Functional redundancy between ANAC019 and ANAC055 was previously described in literature (4,39,56), although some diversity is seen for their senescence-associated regulons (38). Furthermore our results can confirm the presence of binding sites for ANAC055 and ANAC019 in the promoter of *BSMT1*, and the highest target gene overlap (84%) was found between ANAC055 and ANAC019. The functional redundancy of P+COE targets was also evaluated through overlap analysis of enriched functional modules. These functional modules comprise a set of 13 142 genes (1562 modules) annotated with specific functional descriptions based on experimental GO information, protein–protein interaction data, protein–DNA interactions or AraNet gene function predictions (23). As ANAC019 and ANAC055 also show a significant overlap (80%) of functional modules (*P*-value <0.01), these results corroborate the functional redundancy between these two NAC TFs. Other NAC TFs also showed a large overlap in enriched functional modules (Figure 3A and Supplementary Figure S8). Comparing the expression profiles of the different TFs during transcript profiling in different stress conditions (Supplementary Figure S9) further supports the functional overlap between ANAC019, ANAC055, ANAC092, ATAF1 and NAP.

To validate the co-binding of different NAC TFs in close proximity through a palindromic binding site, we systematically screened the promoters of ANAC019, ANAC055 and ANAC092 DE genes for PalNACBSs using the motif CGTN{7-8}ACG (CGT spacer 7 or 8 nucleotides followed by ACG) (42,56). Only 9%, 15% and 12% of the DE genes contained a PalNACBS, and for ANAC019 and ANAC092 this overlap was not significant. Based on the PBM binding data, only 2.2–2.8% of the ANAC019/ANAC055/ANAC092 P+COE target genes are bound by two adjacent NACBSs (spacer of 7 or 8 nucleotides). Considering all NAC TFs, only 3.7% of the P+COE target genes showed this co-binding pattern, corroborating that in most cases NAC binding and regulation is mediated through an individual binding site.

### Overview of functional modules regulated by the different NAC TFs

Apart from comparing the overlap between P+COE genes and DE genes, we also studied the functional landscape of the different TFs using GO and functional modules. Enrichment analysis of P+COE target genes allowed to detect, per TF, the set of modules and associated functions showing significant overlap. The integration of this type of functional data sets can be used to transform the classical GRN into a TF-functional module network from which the diverse functionalities of TFs can be delineated (Supplementary Table S6 and Figure 3). A first set of enriched modules is targeted by multiple TFs (five or more) and is associated with different stress-related functional descriptions as well as signal transduction, transport and secondary metabolism (Figure 3A). The cooperative binding of the genes in these modules mainly comprises known stress-related factors including ANAC019, ANA055, ANAC092 and NAP. The observed association of ATAF1 with growth and development modules is also evident from the vegetative growth phenotypes of plants with perturbed *ATAF1* levels (40). A second set of modules is only targeted by a limited number of TFs and the genes in these modules cover a wider variety of biological processes and molecular functions (Figure 3B). Examples include previously described functions of SND1 and VND7 in cell wall biosynthesis and a role for NTL8 in embryo development (44,45). Furthermore, we found that ANAC092 is linked with multiple transport and signal transduction-related modules, which include known DE genes such as RNS1, ILL6 and MAPKKK19 (43). Of novel relevance to the secondary cell wall-thickening regulator NST2, we highlight genes responding to nutrient starvation and water deficiency (module 10, Figure 3A, Supplementary Table S6), whereas novel target genes of VND7 include genes related to defense and programmed cell death (i.e. MYB TFs), as well those earlier identified genes related to cell wall biogenesis (45). Likewise, a large part of the verified target genes of secondary cell wall regulator SND1 includes genes involved in cell wall biogenesis (i.e. *SND2* and *SND3*) and xylem development (i.e. IRX genes). Furthermore, we highlight the over-representation of functional modules related to transport and senescence to include novel SND1 target genes (Supplementary Table S6). Finally, we observed a striking difference in the presence of genes with conserved motifs between the modules that are targeted by a big number of TFs (>5) and the modules that are targeted by a smaller number of TFs (arrow-head lines in Figure 3A versus B), suggesting that the complexly regulated stress modules represent highly conserved regulatory interactions within plants.

Obviously, the candidate target gene sets together with the associated functional modules offer a promising resource to unravel the functions of the different NAC genes in more detail.

### Using native and synthetic promoter elements to validate PBM results

**Figure 4.**
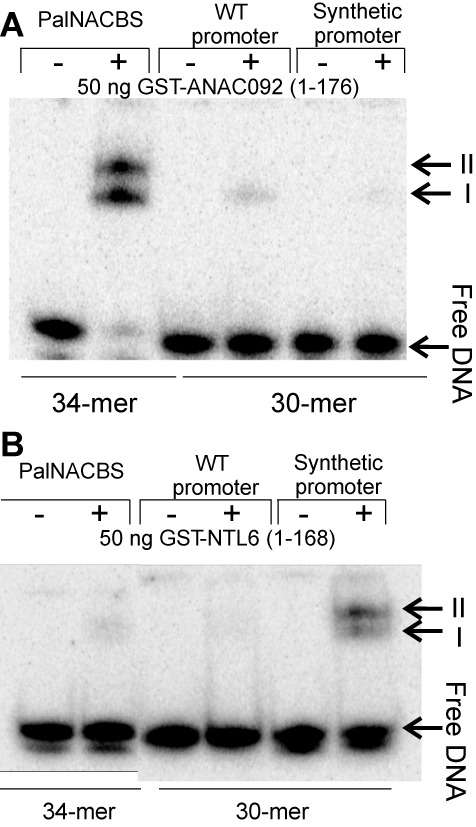
Design of an NTL6 binding site from an ANAC092 promoter. ANAC092 (**A**) and NTL6 (**B**) were tested by EMSAs for binding to a known and validated palindromic NAC-BS consensus (palNACBS), a fragment of an identified ANAC092 target promoter (At1g66390; MYB90) (WT promoter) containing the TACGTCA k-mer and a Synthetic promoter where the same k-mer was mutated to TAaGTaA to mimic an NTL6 binding site.

Binding of TFs to promoter elements is necessary to establish and maintain changes in gene expression levels of target genes (57), and changing the TF-DNA affinity could dramatically affect the regulatory potential of the TF (58). Acknowledging this, we asked whether it would be possible to turn an element present in a target gene promoter identified from our studies into a synthetic promoter element that would both abrogate binding preferences of one TF and direct binding of another TF. Amongst our selected NAC TFs, binding site profiles of ANAC092 are most distantly related (i.e. most divergent PWMs) to the NTL TFs (Figure 1A and Supplementary Figure S10) allowing us to test our hypothesis using these TFs. Firstly, in order to validate our 10-mer PBM data for ANAC092 and NTL6 using EMSA, we used a 30-bp oligonucleotide identical to the promoter of the ANAC092 target gene *MYB90* involved in activating anthocyanin biosynthesis in response to C and N nutrient status (59)*. MYB90* was chosen as it is one of the two genes that passed all filtering tests for ANAC092 (the other one being AT3G02040)(conserved P+COE and DE). The 30-bp oligonucleotide contains a high ES k-mer (TACGTCA.C, 0.46) for ANAC092, yet scores very low for NTL6 (0.02; Supplementary Figure S10). In agreement with our PBM results, our EMSA result shows ANAC092 binding to the 30-bp promoter fragment spanning the −361 bases upstream of the transcription start site of the *MYB90* promoter, whereas no binding was detected using NTL6 (Figure 4). Next, using this oligonucleotide we aimed to turn it into a synthetic NTL6-binding promoter element (*MYB90^Synth^*) using the smallest Levenshtein distance, representing the minimum number of single-nucleotide changes required to change one sequence into another (60). Using this modified 30-bp oligonucleotide, in which TACGTCA was mutated into a high-ES NTL6 target motif (0.47) TAaGTaA, we observed a lowered affinity of ANAC092 for the *MYB90^Synth^* element. This is in accordance with the low-PBM-derived ES value of ANAC092 for TAAGTAA motifs (0.27; Supplementary Figure S10). Most importantly, NTL6 was observed to bind to the *MYB90^Synth^* oligonucleotide with high affinity. As a positive control all proteins were tested for binding to the palindromic NAC-BS consensus (7). Here, ANAC092 showed the strongest affinity. We note that we repeatedly observed two ANAC092-palNACBS and NTL6-*MYB90^Synt^*^h^ complexes. This could potentially arise from binding of two individual dimers to the DNA fragment, also observed in the co-crystal structure of ANAC019-PalNAC BS (36). Taken together, we use a 30-bp oligonucleotide identical to the promoter element of the ANAC092 target gene *MYB90* to validate our PBM data for ANAC092. Also, we report a 2-nucleotide substitution of the ANAC092 binding site lowering the affinity of ANAC092 for this synthetic promoter element and turning it into an NTL6-binding element.

### Using co-expression analysis to uncover the regulatory potential of ANAC092

**Figure 5.**
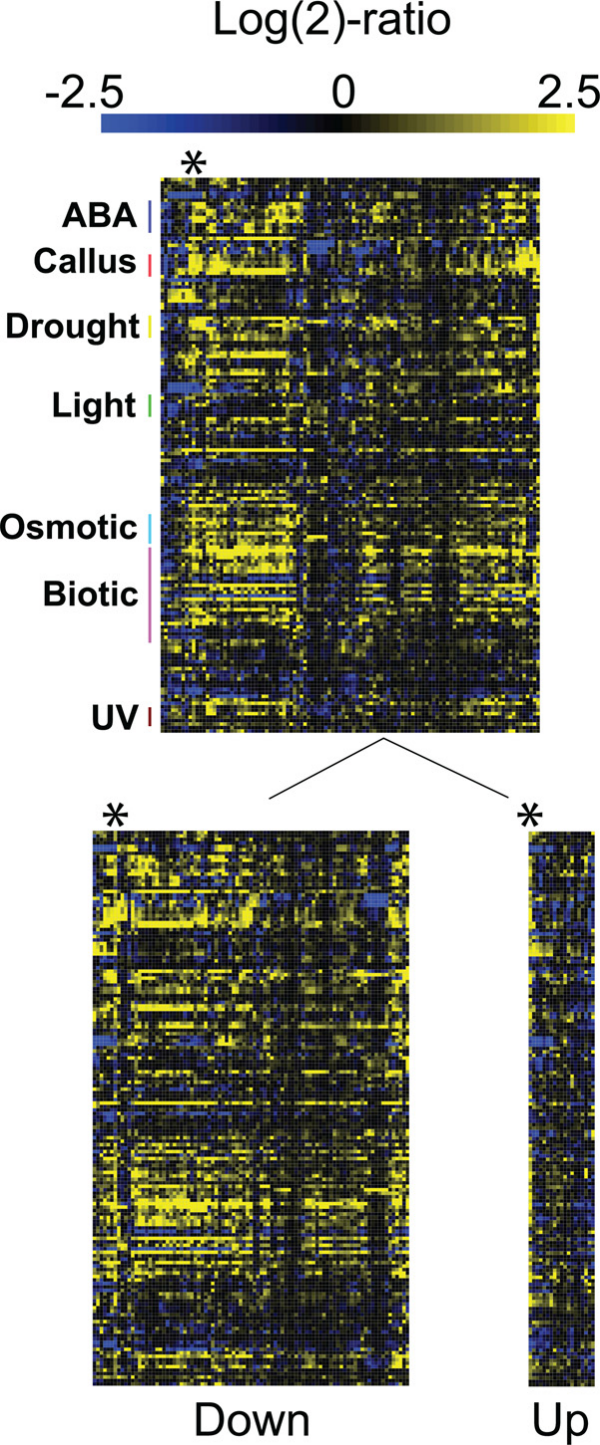
The regulatory potential of ANAC092 is maintained during multiple environmental stresses. Top heatmap displays 107 genes differentially regulated in *anac092* plants compared to Col-0 wild-type plants, all having ANAC092-BS in their 1-kb promoter. Only conditions affecting *ANAC092* expression were included (>2-fold regulation, *P* < 0.05, = 160 perturbations, 705 microarrays). Below, ‘Down’ denotes the 89 genes downregulated in *anac092* mutant plants compared to Col-0 wild-type plants and ‘Up’ denotes the 18 genes upregulated in *anac092* mutant plants compared to Col-0 wild-type plants. * indicates position of ANAC092. To the left, selected conditions perturbing most target genes are highlighted.

Co-expression occurs amongst TFs and target genes (61). To validate our list of putative target genes for our candidate NAC TFs, we hypothesized that genes controlled by individual NAC members should be (i) co-expressed during environmental cues known to affect *NAC* gene expression and (ii) have one or more NAC consensus binding site(s) in their promoter. For this purpose we performed data-mining on >3.000 ATH1 microarray samples from wild-type Col-0 plants, deposited at Genevestigator (37) and, using a stringent (>2-fold regulation, *P* < 0.05) selection criterion for *ANAC092* transcript level perturbations, we found 705 microarray data sets representing 160 perturbations (Figure 5). Using these data, we analysed the co-expression of *ANAC092* and the set of 107 putative target genes. From this analysis we identified two major clusters of genes; those with a positive correlation with *ANAC092* and those with a negative correlation expression pattern compared to *ANAC092*. Interestingly, target genes up-regulated in *anac092* mutant plants almost perfectly match the genes that are downregulated when *ANAC092* is induced. Vice versa, genes downregulated in *anac092* mutants show almost perfect co-expression with *ANAC092*. This indicates that ANAC092 could be both a direct activator and a direct repressor. Moreover, the regulatory potential of ANAC092 is maintained during multiple environmental stresses, and not only during the *anac092* versus Col-0 control condition samples reported by Balazadeh *et al.* (43) that we used in this analysis. The strong *ANAC092* expression perturbations during environmental stresses observed from our analysis are in agreement with the recent results published by Patil *et al.* highlighting *ANAC092*-mediated stress tolerance (62).This result suggests ANAC092 as a TF associated with both positive and negative effects on transcription of a large set of stress-related genes.

## DISCUSSION

A major challenge for predicting gene expression is the accurate characterization and design of genetic circuits that regulate single or multiple genes in response to specific environmental, developmental and physiological cues. In the age of synthetic biology, characterization of TF binding preferences and target gene identification offer major advantages towards engineering genetic circuits for optimal fitness in plant responses towards environmental stresses. However, in order to fully understand the regulatory capabilities of any TF, we need to characterize its DNA-binding specificities with the highest resolution possible in order to minimize erroneous TF–promoter associations resulting in misleading GRNs.

As previously described the CGT[A/G] motif has been identified as the core binding site of stress-inducible NAC TFs (6,7,56). However, this motif present in the DNA-binding sites of cluster 1 is also a core binding site for NAC TFs involved in development and secondary wall synthesis (45). The binding sites of cluster 1 proteins show differences in the flanking regions that mark divergence in the functionality of this cluster's members. These binding differences may be explained by small variations in the DNA-contacting amino acids residues (Supplementary Figure S1B) which, according to the crystallographic model of the ANAC019–DNA complex, are close to the DNA (36). These regions contain both the conserved Arg-88, essential for binding, and the conserved β strand protruding into the major groove of DNA. ANAC019, ANAC055, NAP and ATAF1, which have similar binding sites, constitute a sub-group based on the sequence regions close to DNA (Supplementary Figure S1B), suggesting that these regions influence DNA-binding specificity. These closely related NAC TFs, however, also show different preferences for A/G of the core binding site which is not easily explained from the sequence alignment. SND1, NST2, VND3 and VND7 involved in secondary wall synthesis (44,45) cluster together (Supplementary Figure S1A) (13) yet the DNA-binding specificities of SND1 and NST2 are closer to those of ANAC092 than those of VND3 and VND7. This is unexpected considering that the expected DNA contacting residues for all these TFs are identical. Further analysis will reveal if substitution of single amino acid residue, such as the change of a conserved basic residue to a glutamine (position 127 of VND3), possibly in contact with DNA (36), may affect DNA-binding specificity.

Surprisingly, and in contrast to reports showing that binding of NTL6 to the PR genes depends on the NAC-BS core (48) NTL6 and NTL8 do not recognize sequences with the NAC-BS core. We did not observe any overlap between DNA specificities of clusters 1 and 3, leading to the hypothesis that these proteins, whilst members of the same general TF family, are functionally divergent from their paralogues. As seen in bHLH and homeodomain proteins, few amino acids can play a critical role in the definition of DNA specificities for single TFs (15,16,18,63). Indeed, as few as five positions show differences between NTL6, NTL8 and the remaining NAC proteins. These are at positions NTL6 74 (Y->F), 102 (R->K), 116 (R->K), 121 (H->Y) and 130 (R->K), with 121 (H->Y) representing the chemically most significant change (Supplementary Figure S1B). Whilst positions 116, 121 and 130 are close to DNA, we cannot rule out that positions 74, 102 and additional regions may also influence specificity of these NAC proteins. Although single amino acid residues may dictate DNA-binding specificity, conformational changes of, for example, the DNA-contacting NAC loops (36) may also influence DNA-binding specificity (64). Clearly, further structural analyses are needed to identify the fine molecular determinants of NAC-DNA-binding specificity and affinity even though these presented data can be sufficient to estimate DNA specificities for NAC proteins in terms of cluster 1, 2 or 3.

NAC binding sequences selected in some other studies are palindromic sets of two adjacent sites reflecting that NAC TFs form and bind DNA as dimers (7,11). However, as seen in this study, single NACBSs can be sufficient for NAC promoter binding. This effect has also been shown to be true from the analysis of ANAC072/019/055 binding to the *ERD1* promoter (56), ANAC096 binding to the *RD29A* promoter (14) and ATAF1 binding to the *9-cis-epoxycarotenoid dioxygenase* (*NCED3*) promoter (40). In fact, the single ATAF1 binding site identified by PBM analysis was used to identify *NCED3* as a direct ATAF1 target gene (40). The fact that a single NAC-BS is sufficient for NAC binding is also supported by *in vitro* analysis showing that although NAC dimerization is needed for detectable DNA binding, only a single NACBS is needed for binding (7). Furthermore, a recent DNase I footprint of ANAC019 and the palindromic PalNAC BS showed asymmetric protection (i.e. saturation) of the two single binding sites in the palindrome. (36). Despite this, heterodimerization of NAC TFs (11) may expand the DNA-binding specificity spectrum *in vitro*, as suggested for the bHLH TFs (15,16). This variability between single or double binding sites can bring yet another level of genetic regulation in NAC-dependent stress response in *A. thaliana*. It is plausible that promoters showing palindromic dimer sites could be differentially regulated by combinations of NAC homo- and hetero-dimers thus expanding on the range of stress signals recognized. To better understand this process a large-scale NAC dimerization screen followed by NAC dimer DNA-binding studies would be required.

A major challenge for the characterization of GRNs using high-throughput TF binding data is to properly translate DNA specificities in meaningful lists of potentially regulated genes. Transcription-factor binding affinities determined *in vitro* have been shown to quantitatively predict the output of complex target promoters (15,65) yet the risk of contaminating the target detection analysis with false positives and false negatives is a real threat. By integrating different layers of evidence, such as co-expression information, differential expression in mutant plants, motif conservation and functional gene modules, we were able to obtain meaningful and accurate functional predictions for the studied TFs, including the verification of 31 previously identified NAC TF target genes. This emphasizes the applicability of our workflow using PBM and functional modules to uncover NAC TF target genes. The improved specificity obtained through the integration of complementary functional genomics data sets is in agreement with recent observations from genome-wide chromatin immunoprecipitation experiments, where typically only a minor fraction of bound regions corresponds with bona-fide-regulated target genes (66). As a consequence, also for ChIP-chip and ChIP-Seq experiments, detailed motif and expression information are required to define an accurate set of functional *in vivo* target genes.

Due to the fact that NAC TFs have a large potential in plant engineering and production of more ‘robust’ economically important crops (6,9,67) detailed knowledge about TF–DNA interfaces and target gene perturbations become crucial knowledge for the exploitation of rationally designed GRNs for improved stress tolerance and other economically important traits. As shown here, the minimal changes in NACBS required to engineer, and potentially redirect, single TF GRNs can hold interesting solutions for future breeding and genome editing projects. For instance, identification of SNPs in TF-BSs of putative orthologous gene promoters related to certain morphological traits can be harnessed for improving or abrogating TF DNA-binding affinity and thereby transcriptional output. Further away, specific Cas9-based genome editing (68) could be applied to balance transcriptional output to specific environmental conditions using a one-TF-many-target-genes approach. Using the knowledge and information obtained from this study, we could envision modifying specific NACBSs, with great accuracy, to rewire GRNs with the final aim at improving or generate *de novo* stress responses in *A. thaliana* and other plants. This novel GRN design could lead to the generation of drought or other climatic-stress resistant crops, which could be designed to contrast desertification and the resulting loss in food production.

## ACCESSION NUMBERS

Sequence data from this article can be found in TAIR (The Arabidopsis Information Resource) and EMBL (European Molecular Biology Laboratory) data libraries using the nomenclature names, synonyms and accession numbers in Supplementary Table S1.

## SUPPLEMENTARY DATA

Supplementary Data are available at NAR Online, including [69–73].

SUPPORTING INFORMATION
