# Supplementary material for: A DNA-binding-site landscape and regulatory network analysis for NAC transcription factors in Arabidopsis thaliana
Source: Nucleic Acids Res. 2014 Jun 9;42(12):7681–93. doi: 10.1093/nar/gku502 (PMC4081100; doi:10.1093/nar/gku502)
Supplement: SUPPORTING INFORMATION [file supp_42_12_7681__index.html]

A DNA-binding-site landscape and regulatory network analysis for NAC transcription factors in Arabidopsis thaliana — SUPPORTING INFORMATION 

# A DNA-binding-site landscape and regulatory network analysis for NAC transcription factors in *Arabidopsis thaliana*

## SUPPORTING INFORMATION

**Files in this Data Supplement:**

- Supplemental Information I
- Supplemental Information II
