# Supplementary material for: A DNA-binding-site landscape and regulatory network analysis for NAC transcription factors in Arabidopsis thaliana
Source: Nucleic Acids Res. 2014 Jun 9;42(12):7681–93. doi: 10.1093/nar/gku502 (PMC4081100; doi:10.1093/nar/gku502)
Supplement: SUPPORTING INFORMATION [file supp_gku502_nar-00465-z-2014-File007.pdf]

# Lindemose *et al.* Supp. Document 1

**Selection of NAC TFs for PBM analysis**

The number of known NAC binding targets is still relatively limited (6, 41). In this study, a systematic analysis of the DNA-binding specificity of 14 NAC TFs was performed. ANAC019 was selected because the tertiary structure of its NAC domain is known (7,11) and because it is implicated in networks of stress responses and senescence (38, 56). ANAC055 is functionally and structurally closely related to ANAC019, and ATAF1 is a NAC positive control for PBM experiments (38, 56, 2, 13, 40, 39) (Figure 1A). These NAC TFs and senescence-associated NAP (41) also cluster together based on gene regulation by different plant hormones (42) and they bind to the same sequence in the *ERD*1 promoter (56, 69). Therefore, PBM analysis of these could reveal simple relationships between NAC amino acid sequence and DNA-specificity. ANAC092/ORE1 was selected to analyze further for DNA-binding specificities associated with senescence (43). Another group of closely related NAC TFs, represented by VND3, VND7, NST2 and SND1, is central to secondary cell wall formation and has been studied extensively with respect to phenotypes and target genes (44-46). All of these proteins show small evolutionary distances (Figure 1A) and a high degree of sequence similarity (Figure 1B) for the region involved in DNA binding (36).

NTL8 and NTL6 are membrane-bound NAC TFs that can act through direct binding to a conserved sequence in *Pathogenesis-Related* (*PR*) genes (47,71,72) allowing comparison of the PBM and *in vivo* promoter binding data. Interestingly, these proteins show marked sequence differences both at the distance and at sequence identity levels with the other NAC proteins (Figure 1A and 1B) yet they clearly are members of the NAC family (2).

This study also included three distantly related NAC proteins (Figure 1A and B): SOG1 (49), ANAC003 and VOZ2. VOZ2 has an unconventional CCCH Zinc finger region, known as the VOZ (Vascular plant One-Zinc finger) domain, at the N-terminal end of the NAC domain (2, 50, 73).

NAC TFs are characterized by the N-terminal DNA-binding NAC domain and various intrinsically disordered C-terminal domains which function as transcriptional regulatory domains (TRDs)(2) (Supp. Figure S1). Considering that only the NAC domains of the 14 TFs is used in this study and since remote disordered regions may fine tune both specificity and affinity of DNA-binding (51) full-length ANAC092 was also used for the PBM experiments. Finally, the WRKY domain of the WRKY1 TF was included in the study to bench mark our data to its well-defined DNA-binding specificity (52).

69. Olsen, A.N., Ernst, H.A., Leggio, Lo, L. and Skriver, K. (2005) DNA-binding specificity and molecular functions of NAC transcription factors. Plant Sci., 169, 785–797.

70. Xue, G.P., Bower, N.I., McIntyre, C.L., Riding, G.A., Kazan, K. and Shorter, R. (2006) TaNAC69 from the NAC superfamily of transcription factors is up-regulated by abiotic stresses in wheat and recognises two consensus DNA-binding sequences. Funct. Plant Biol., 33, 43–57.

71. Seo, P.J., Kim, S.G. and Park, C.M. (2008) Membrane-bound transcription factors in plants. Trends Plant Sci, 13, 550–556.

72. Kim, S.G., Lee, S., Seo, P.J., Kim, S.K., Kim, J.K. and Park, C.M. (2010) Genome-scale screening and molecular characterization of membrane-bound transcription factors in Arabidopsis and rice. Genomics, 95, 56–65.

73. Yasui, Y., Mukougawa, K., Uemoto, M., Yokofuji, A., Suzuri, R., Nishitani, A. and Kohchi, T. (2012) The Phytochrome-Interacting VASCULAR PLANT ONE-ZINC FINGER1 and VOZ2 Redundantly Regulate Flowering in Arabidopsis. Plant Cell, 10.1105/tpc.112.101915.

# Lindemose *et al.* Supp. Fig. S1

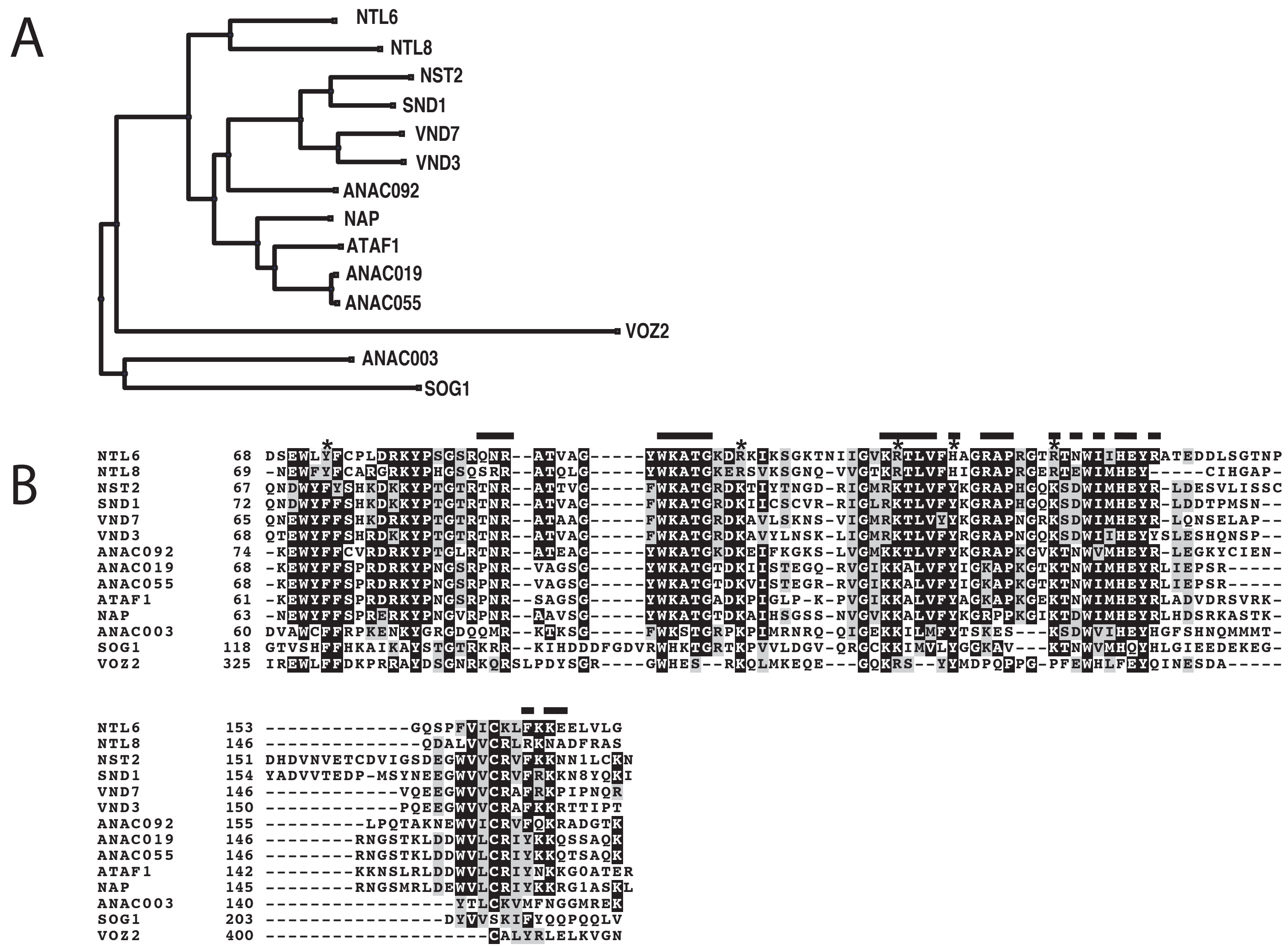


**Supp. Figure S1 – Sequence conservation and identity of NAC protein DNA binding domains.**

A) NAC DNA binding domain sequence similarity tree for all studied NAC proteins shows 3 main clusters for our candidate TFs. Cluster I contains ANAC092, NST2, SND1, VND3, VND7, ANAC019, ANAC055, ATAF1 and NAP; Cluster II contains NTL6 and NTL8 and, finally, Cluster III contains VOZ2, ANAC003 and SOG1.

B) Multiple sequence alignment of the DNA binding regions of the selected 14 NAC proteins. Residues that based on the x-ray model of the ANAC019-DNA complex are close to DNA are shown by a bar. Residues marked with black boxes are common to at least half of the sequences and residues marked in grey boxes are chemically similar in half of the sequences. Asterisks highlight those residues showing remarkable divergence between NTL6, NTL8 and the remaining NAC proteins.

Lindemose *et al.* Supp. Fig. 2

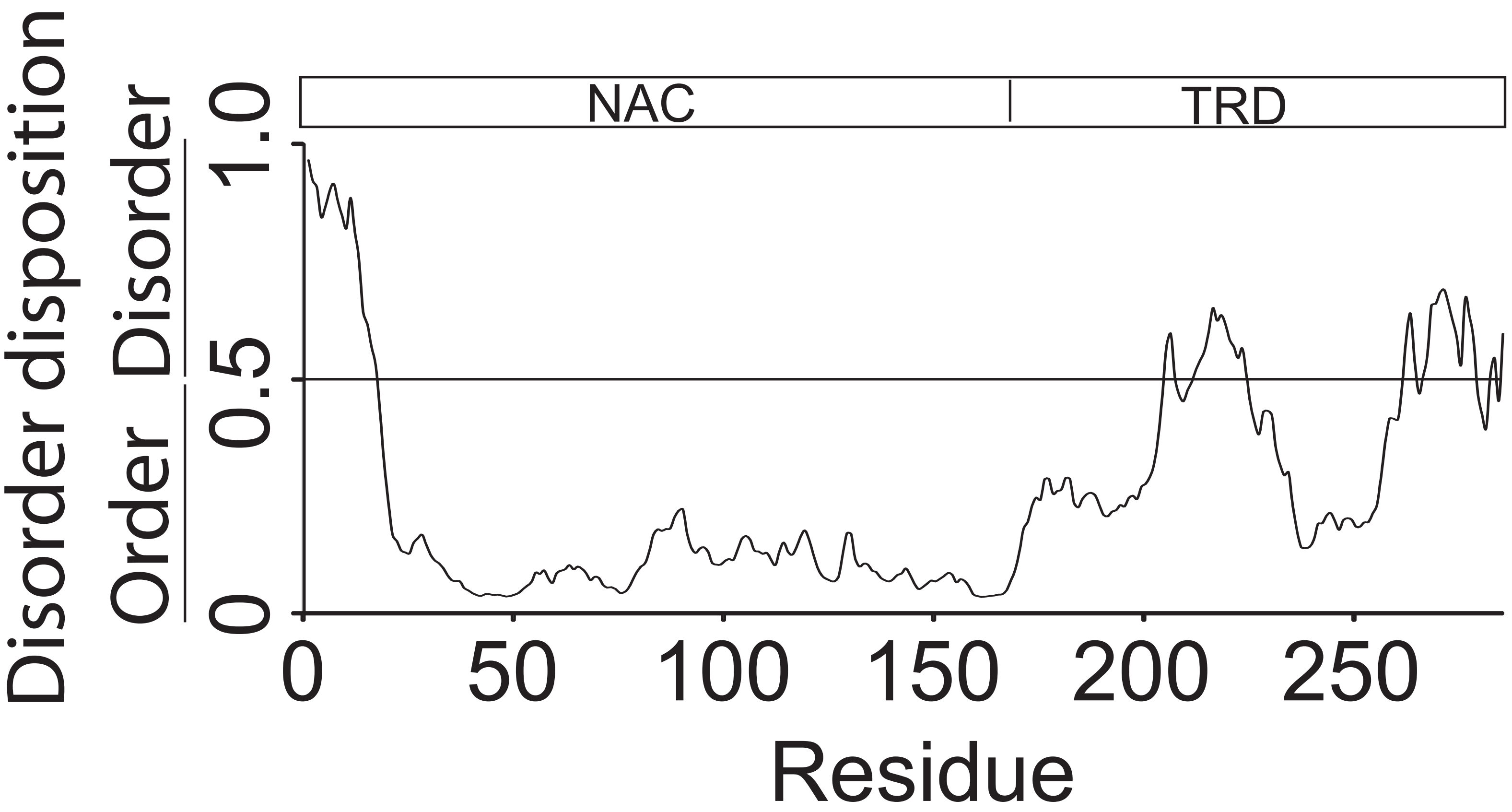


**Supp. Figure S2 - Disorder prediction in ANAC092.**
ID prediction of ANAC092 was performed using PONDR-FIT (Xue et al. 2010). A threshold is applied with disorder assigned to values ≥ 0.5.

# Lindemose *et al.* Supp. Figure 3

**A**

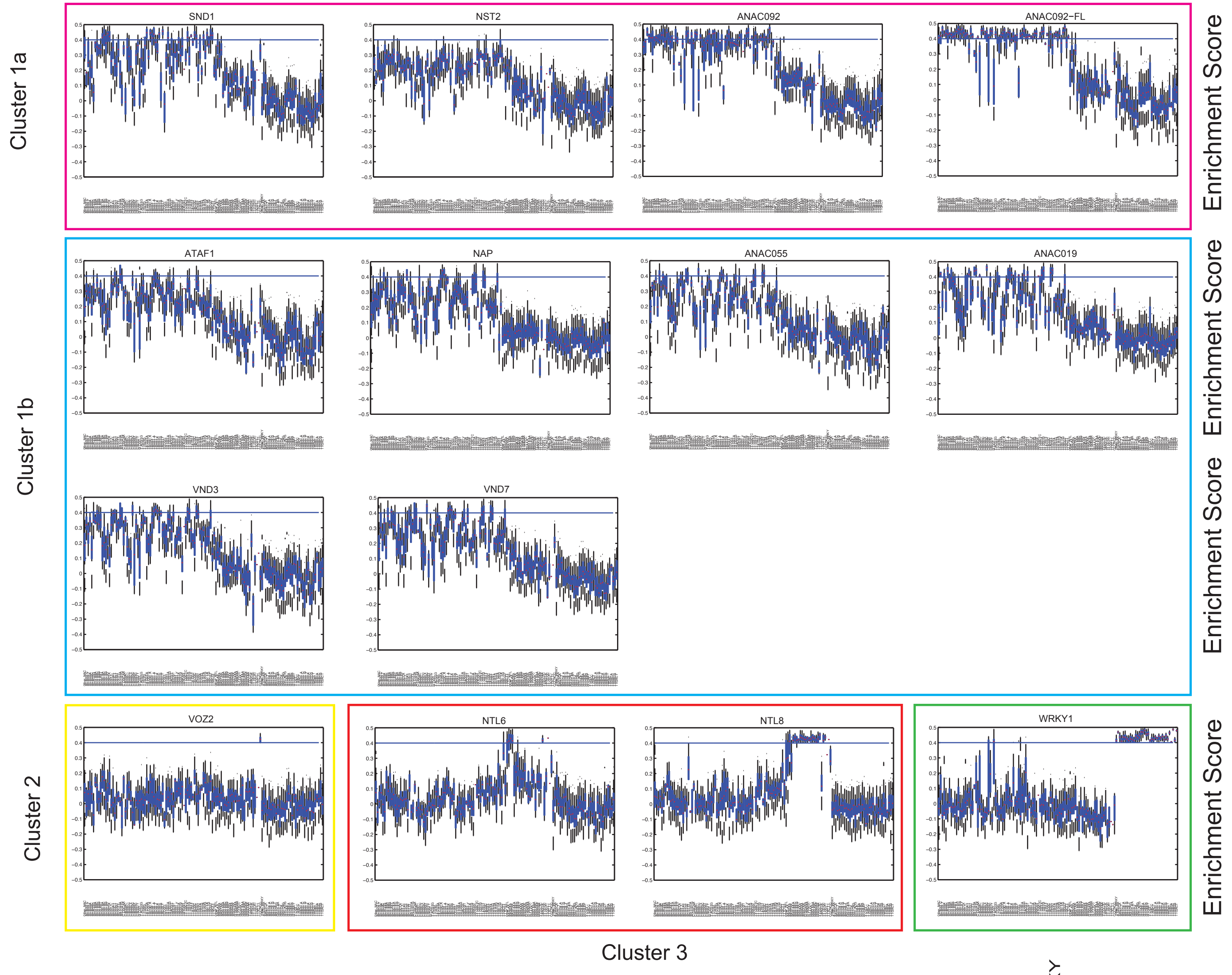


**B**

0ther_NAC
ACACG.C
ACACG.T
ACACGA
ACACGC
ACACGG
ACG.AAA
ACG.AAC
ACG.AAG
ACG.AAT
ACGC.AA
ACGC.AC
ACGC.AG
ACGC.AT
ACGCTA
ACGT.T
ACGTAT
ACGTCT
ACGTGT
ACGTTT
AG.CGTA
AG.CGTG
C.CGCAA
CACGAC
CACGC.
CACGCC
CACGCT
CACGGC
CACGGT
CACGTC
CACGTT
G.CGT
T.ACGTG
T.CCGT
T.CGTD
TA.CGTA
TA.CGTG
TACGC.A
TACGT
TACGT.A
TACGT.T
TACGTA
TACGTC
TACGTG
TACGTT
TCCGT
TG.CGT
TG.CGTA
TG.CGTG
TGACGT
TGCCGT
TGCGT
TGCGT.Y
TGCGTA
TGCGTG
TGGCGT
TGTCGT
TT.CGCTC
TT.CGTA
TT.CGTG
TT.CGTT
TTA.CGT
TTACGC
TTACGT
TTCCGT
TTCGTA
TTCGTG
TTG.GTA
TTG.GTG
TTGC.TA
TTGC.TG
TTGCGT
TTGCTT
TTTCGT
0ther_NTL
AAG.AA
AAGGAA
AAGTAA
AGG.AAA
AGG.AAC
AGG.AAG
AGG.AAT
AGGAAA
AGGGAA
AGGTAA
G.GGAAA
G.GGGAA
G.TTTCC
GGAAA
GGG.AAA
GGG.AAC
GGGGAA
GGGTAA
T.CCCTC
TAAGTA
TT.CCCC
TT.CCTC
TTCGT
TTTC.TT
CCCNCC
.0ther_WRKY
A.GTCAA
AAGTCA
AGTC.T
AGTCA.A
AGTCA.C
AGTCA.G
AGTCA.T
AGTCAG
GGTC.AA
GGTC.T
GGTCA.
GGTCAG
GTC.ACG
GTCAA.
GTCAAA
GTCAAC
GTCAAG
GTCAAT
T.CGT
T.GACC
T.GACT
TGACC.A
TGACC.C
TGACC.G
TGACC.T
TGACCA
TGACCC
TGACCG
TGACCT
TTGAC.
TTGACA
TTGACC
TTGACG
TTGACT

**Supp. Figure S3 - A)** Boxplots of ES distributions for 130 signature 6-mers for all tested TFs. TF boxplots are grouped according to clusters in Figure 1A. **B)** List of the 130 key 6-mers describing NAC DNA specificities as presented on the x-axis of all boxplots

# Lindemose *et al.* Supp. Figure S4

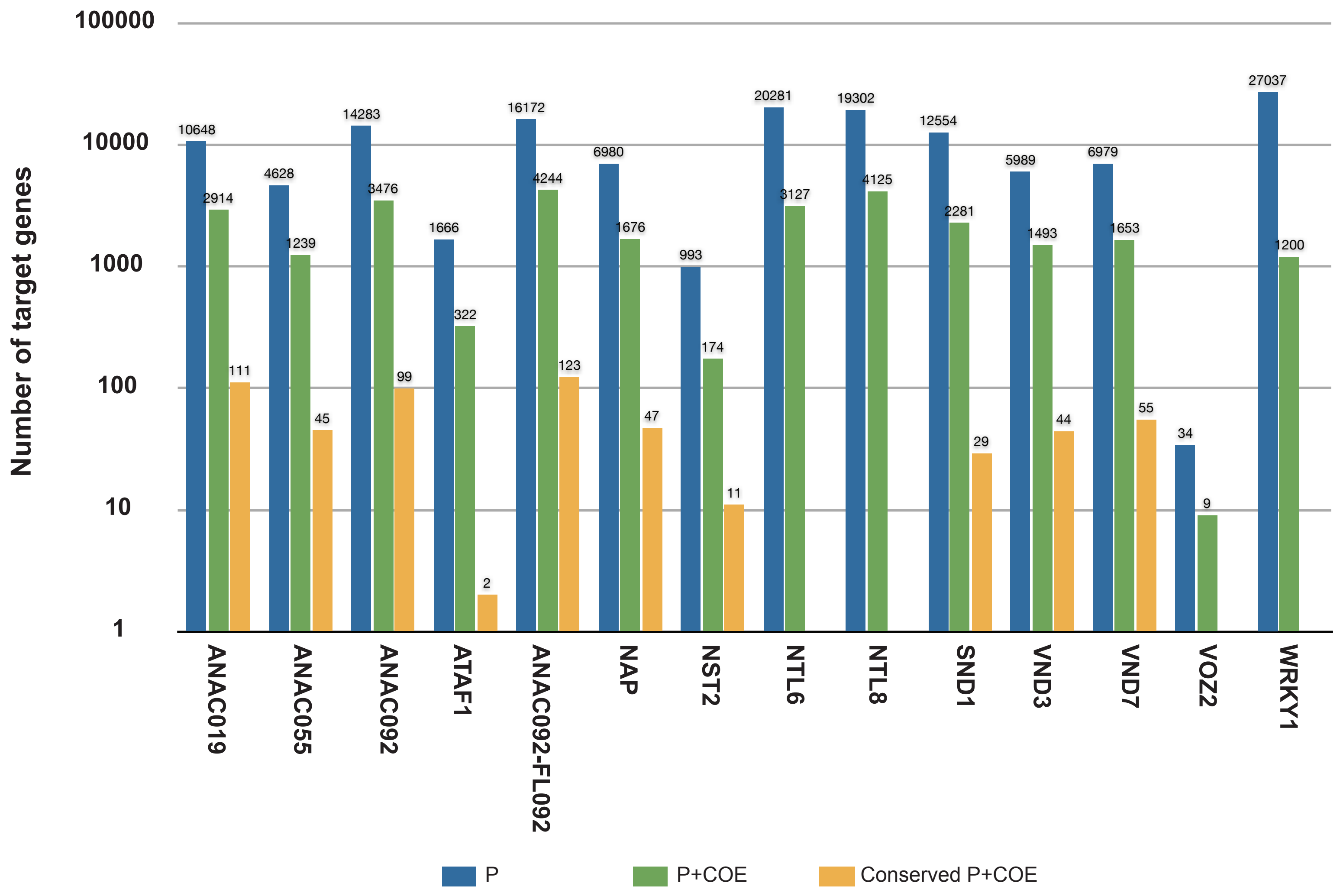


**Supp. Figure S4. – Overview of the number of target genes for different NAC TFs and filtering approaches.**
The number of target genes is shown for each TF. Blue bars indicate the number of P target genes through simple screening of promoters with high scoring k-mers. Green and yellow bars show the number of target genes when integrating co-expression information and conserved motif information, respectively.

# Lindemose *et al.* Supp. Figure S5

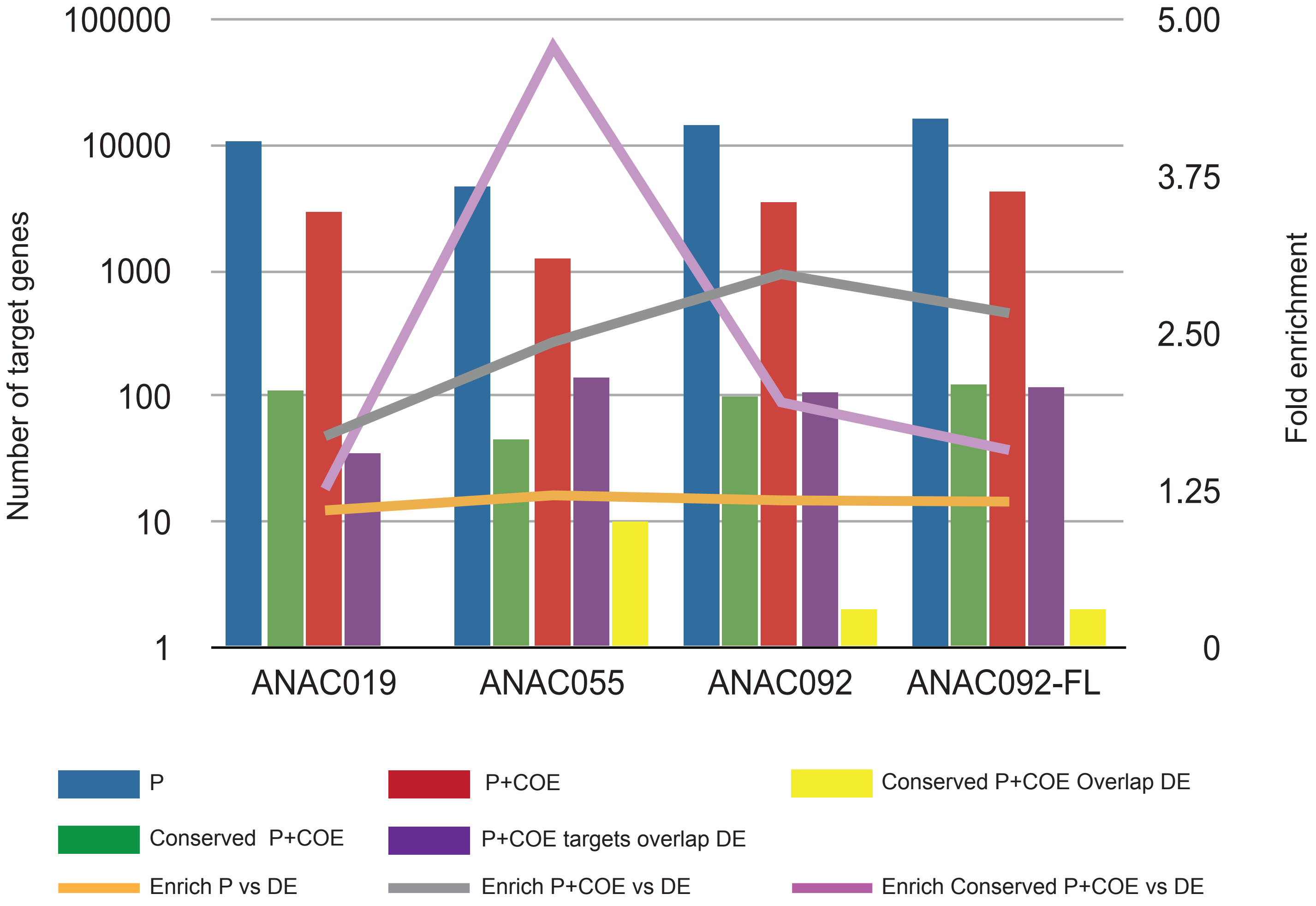


**Supp. Figure S5 – Overlap analysis of different sets of PBM target genes and enrichment analysis for differentially expressed genes.** Bars show the number of target genes for different PBM filtering approaches. COE and Conserved refer to the integration of co-expression and motif conservation information, respectively. DE refers to integration of differentially expressed gene sets. Purple, grey and yellow lines display enrichment for DE genes for P targets, integration with co-expression and integration with conserved motif information, respectively.

# Lindemose *et al.* Supp Figure S6

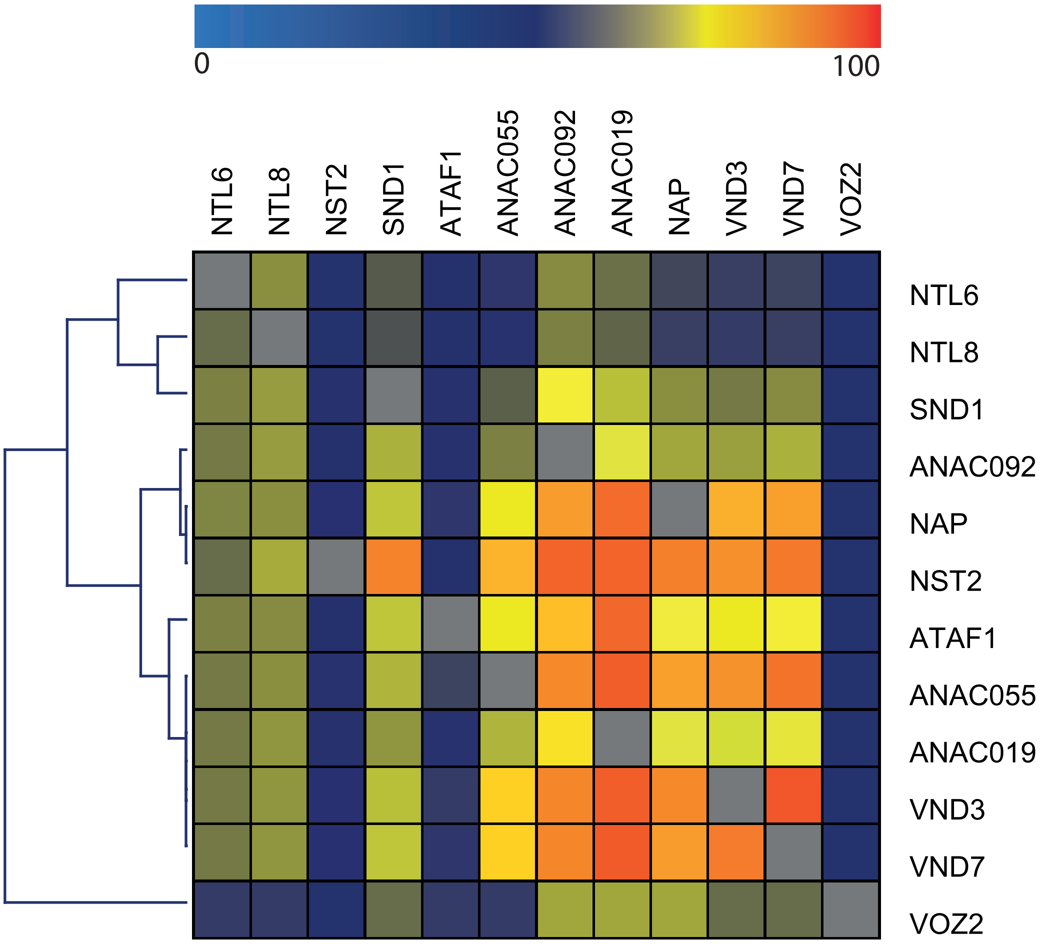


**Supp. Figure S6 – Clustering of NAC TFs based on shared target genes.**
The overlap between P+COE target genes for all TFs was measured and clustered using hierarchical clustering with complete linkage (with Pearson correlation as a distance metric). Overlap was defined as the fraction of target genes for TF row shared with TF column and ranges from 0 to 100%.

# Lindemose *et al.* Supp. Figure S7

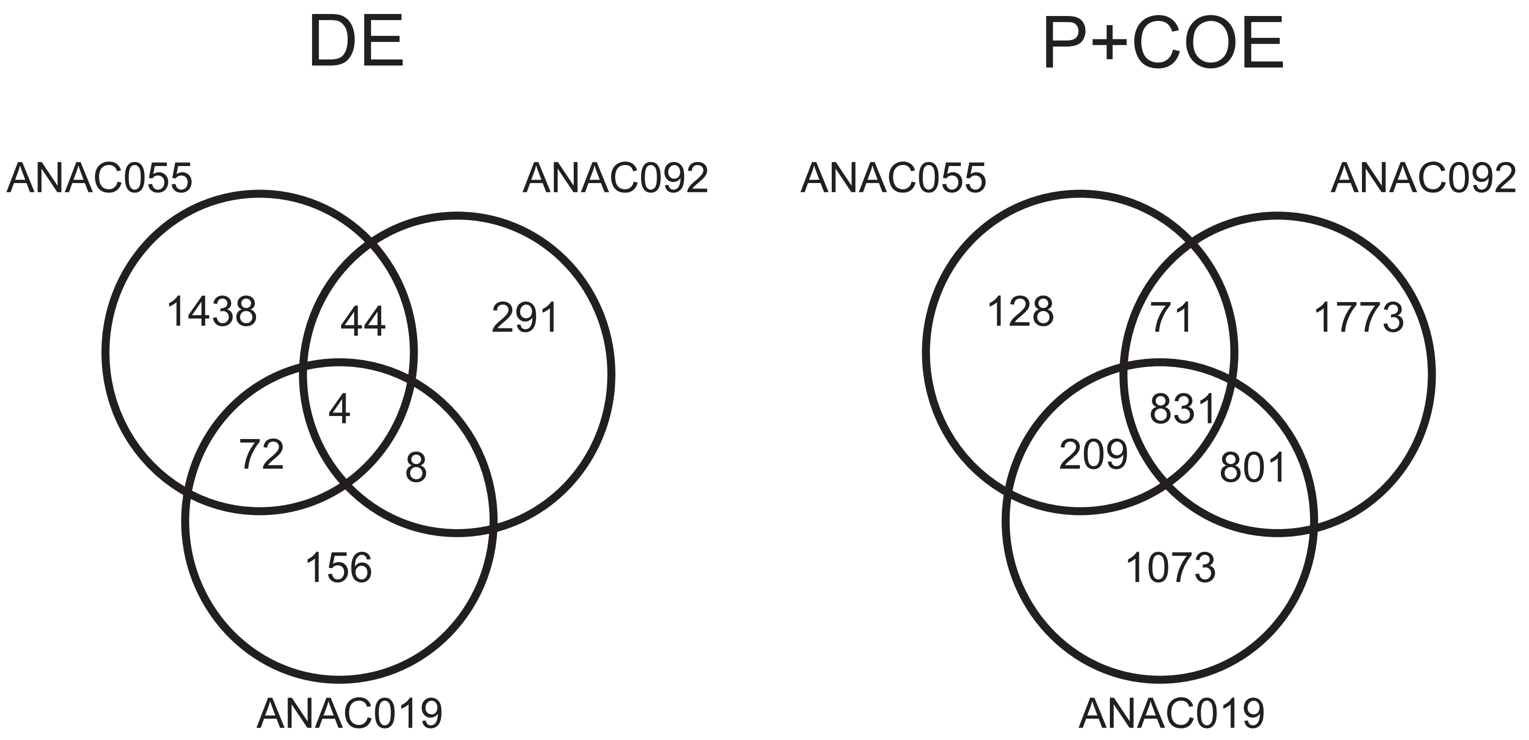


**Supp. Figure S7 – Overlap analysis of DE genes and P+COE target genes for ANAC055, ANA092 and ANAC019.** Overlap between DE genes and P+COE target genes illustrates the redundancy in target genes that exists between these TFs. P+COE refers to predicted target genes combined with co-expression information.

# Lindemose *et al*. Supp. Figure S8

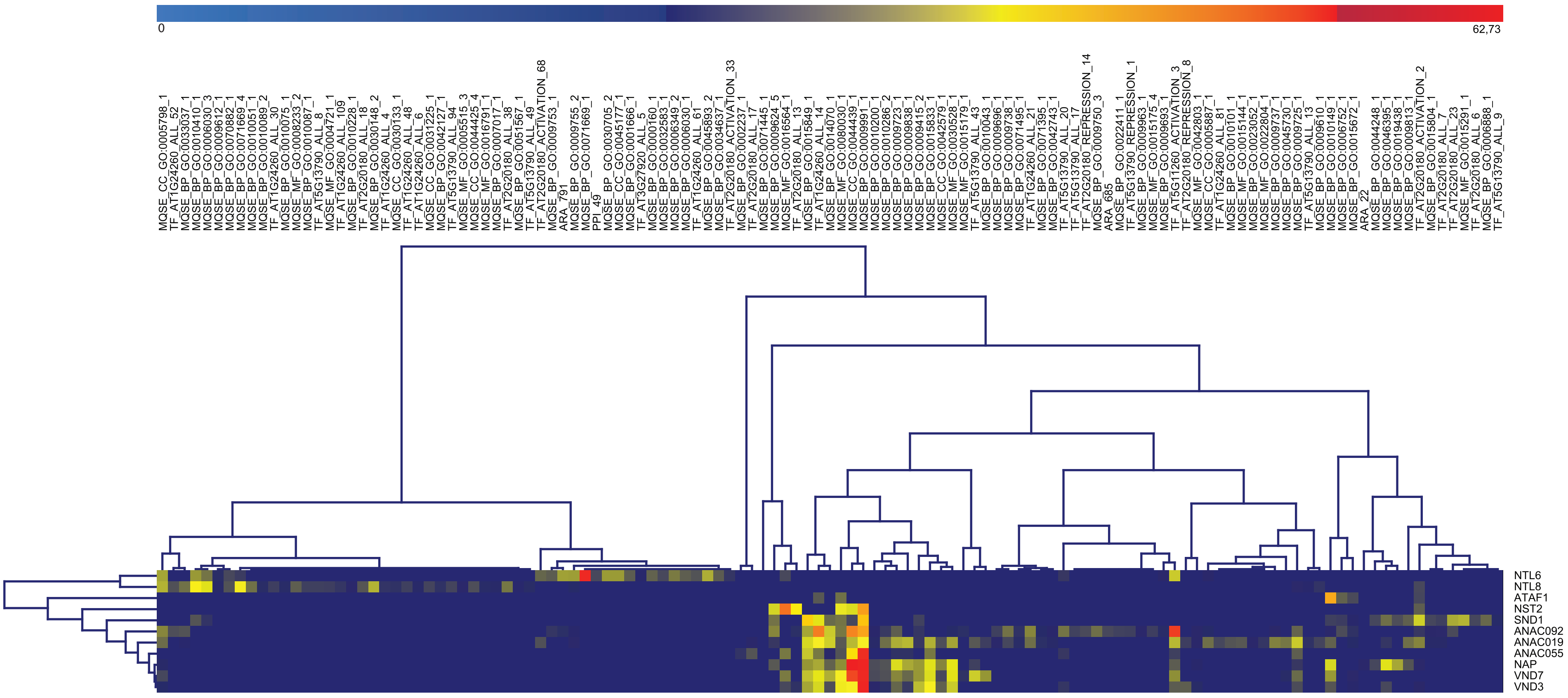


**Supp. Figure S8 – Clustering of NAC TFs based on shared target gene modules.**
Based on the enriched modules per TF (P+COE target genes), hierarchical clustering was performed on both TFs and modules. Enrichment folds are displayed as different colors in the heatmap.

# Lindemose *et al.* Supp. Figure S9

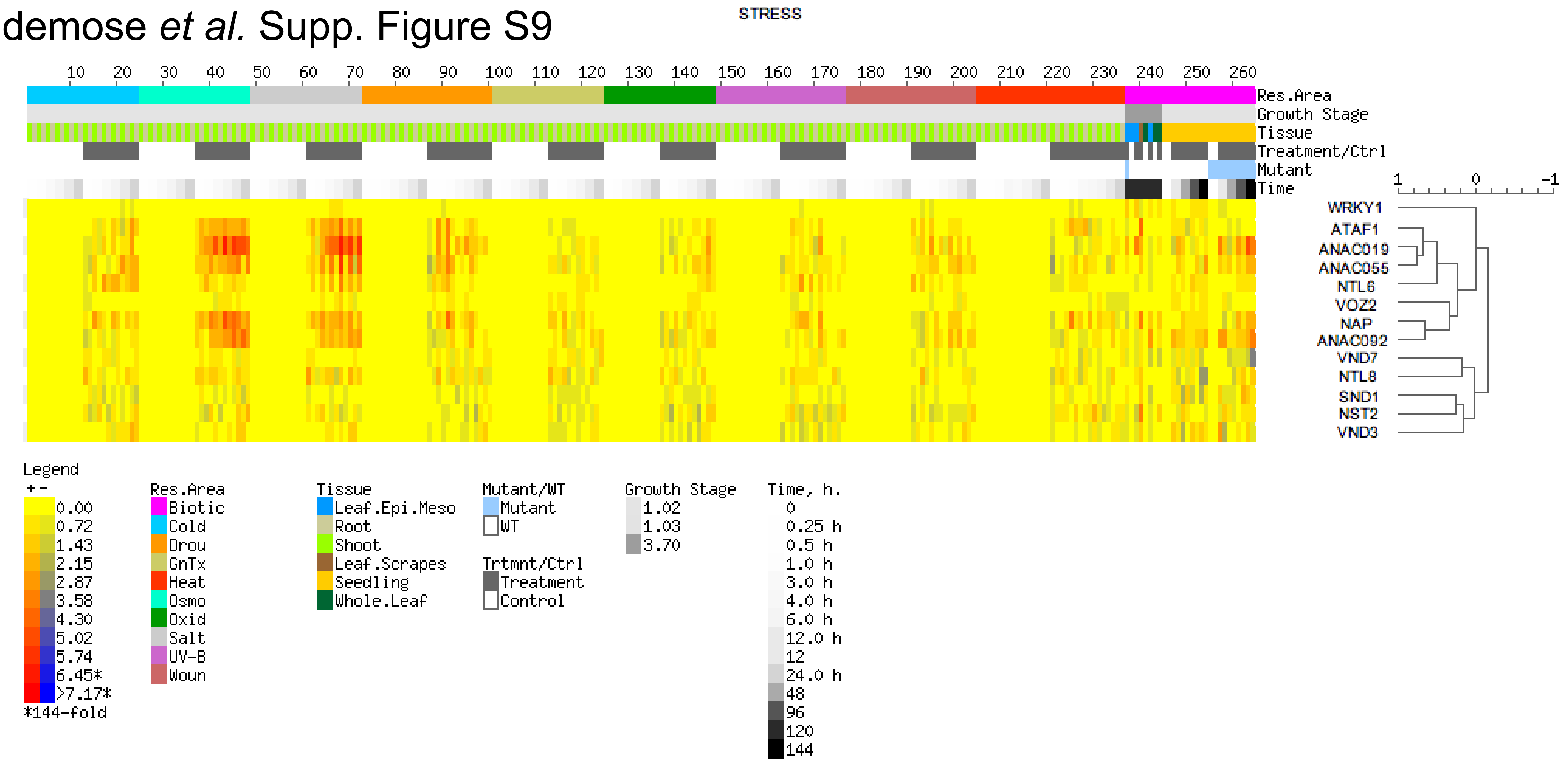


**Supp. Figure S9 – Overview of stress-responsive expression profiles for the different NAC TFs.**
Expression levels in different stress conditions were clustered for all TFs using the BAR Toronto Expression Browser (Stress series).

# Lindemose *et al.* Supp Figure S10

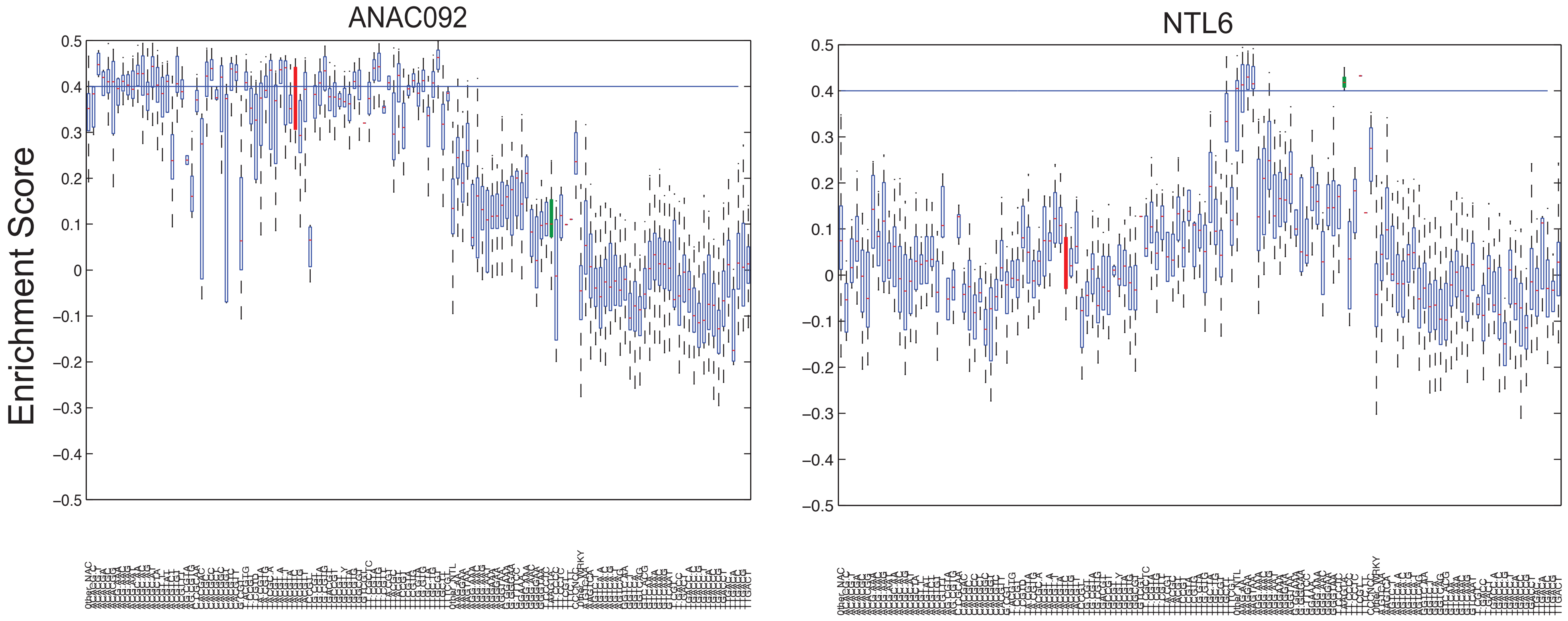


**Supp. Figure S10 ANAC092 and NTL6 show non overlapping k-mer signatures.**
For each protein, the red box shows the ES distribution of k-mers containing the TACGTC key k-mer, which is specific for Cluster 1a and 1b proteins. The green box shows the ES distribution of k-mers containing the TAAGTA key k-mer, specific for Cluster 3 proteins.
